# Supplementary material for: Estimation of the fraction of COVID-19 infected people in U.S. states and countries worldwide
Source: PLoS One. 2021 Feb 8;16(2):e0246772. doi: 10.1371/journal.pone.0246772 (PMC7869996; doi:10.1371/journal.pone.0246772)
Supplement: S1 Fig — (A) Expectation-maximization (EM) iteration to update latent time courses involved in actual infections. (B) Workflow of initialization, EM iterations, and calculation of confidence intervals. (PDF) [file pone.0246772.s001.pdf]

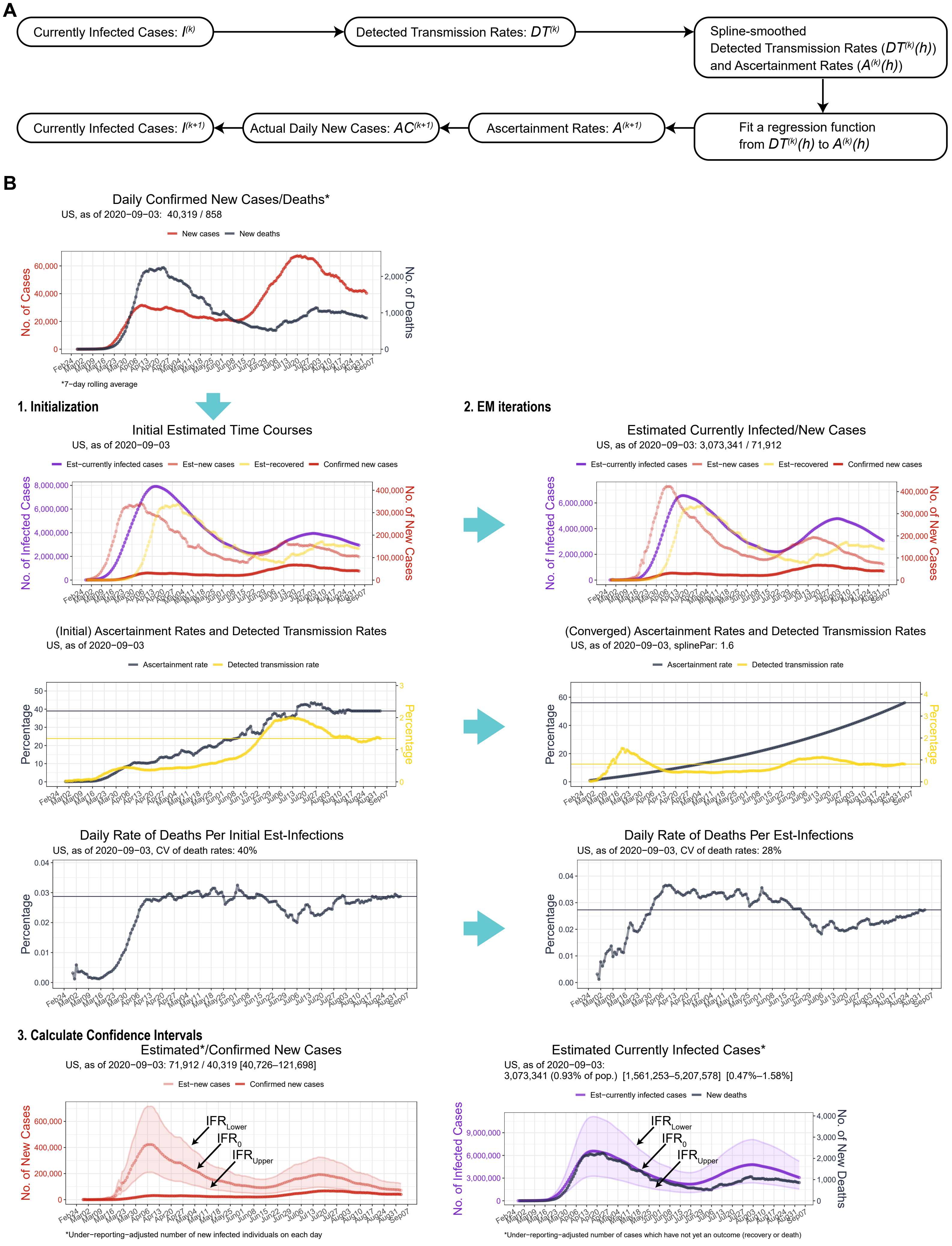
A
Currently Infected Cases: I^(k)
Detected Transmission Rates: DT^(k)
Spline-smoothed
Detected Transmission Rates (DT^(k)(h))
and Ascertainment Rates (A^(k)(h))
Fit a regression function
from DT^(k)(h) to A^(k)(h)
Ascertainment Rates: A^(k+1)
Actual Daily New Cases: AC^(k+1)
Currently Infected Cases: I^(k+1)
B
Daily Confirmed New Cases/Deaths*
US, as of 2020−09−03: 40,319 / 858
New cases
New deaths
No. of Cases
No. of Deaths
*7−day rolling average
1. Initialization
Initial Estimated Time Courses
US, as of 2020−09−03
Est−currently infected cases
Est−new cases
Est−recovered
Confirmed new cases
No. of Infected Cases
No. of New Cases
2. EM iterations
Estimated Currently Infected/New Cases
US, as of 2020−09−03: 3,073,341 / 71,912
(Initial) Ascertainment Rates and Detected Transmission Rates
US, as of 2020−09−03
Ascertainment rate
Detected transmission rate
Percentage
(Converged) Ascertainment Rates and Detected Transmission Rates
US, as of 2020−09−03, splinePar: 1.6
Daily Rate of Deaths Per Initial Est-Infections
US, as of 2020−09−03, CV of death rates: 40%
Daily Rate of Deaths Per Est-Infections
US, as of 2020−09−03, CV of death rates: 28%
3. Calculate Confidence Intervals
Estimated*/Confirmed New Cases
US, as of 2020−09−03: 71,912 / 40,319 [40,726–121,698]
Est−new cases
Confirmed new cases
No. of New Cases
IFR_Lower
IFR_0
IFR_Upper
*Under−reporting−adjusted number of new infected individuals on each day
Estimated Currently Infected Cases*
US, as of 2020−09−03:
3,073,341 (0.93% of pop.) [1,561,253–5,207,578] [0.47%–1.58%]
Est−currently infected cases
New deaths
No. of Infected Cases
No. of New Deaths
*Under−reporting−adjusted number of cases which have not yet an outcome (recovery or death)
