# Supplementary figures and images for: Estimation of the fraction of COVID-19 infected people in U.S. states and countries worldwide
Source: PLoS One. 2021 Feb 8;16(2):e0246772. doi: 10.1371/journal.pone.0246772 (PMC7869996; doi:10.1371/journal.pone.0246772)

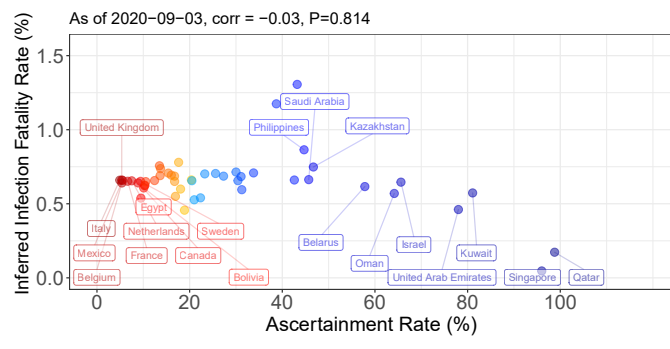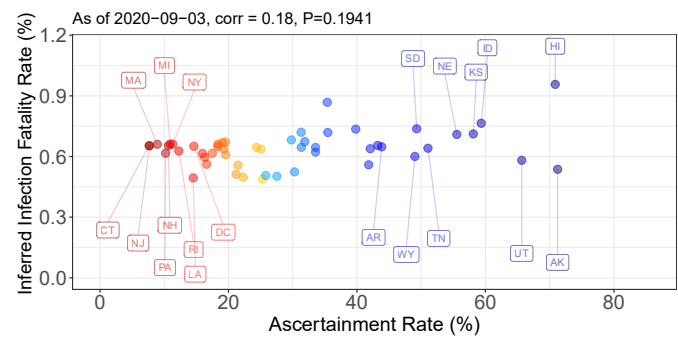

Supplement: S2 Fig — Scatter plots between the inferred infection-fatality-rates (IFR) and the whole period ascertainment rates for the 50 countries (left) and 50 U.S. states (right). The inferred IFR is the ratio of total confirmed deaths to the under-reporting-adjusted total number of cases on a date 18-day before, accounting for the mean duration from infection to death. Spearman rank correlations and their P-values are shown. (PDF) [file pone.0246772.s002.pdf]
