# Supplementary material for: Estimation of the fraction of COVID-19 infected people in U.S. states and countries worldwide
Source: PLoS One. 2021 Feb 8;16(2):e0246772. doi: 10.1371/journal.pone.0246772 (PMC7869996; doi:10.1371/journal.pone.0246772)
Supplement: S1 Appendix — (PDF) [file pone.0246772.s003.pdf]

S1 Appendix for

Estimation of the fraction of COVID-19 infected people in U.S. states and countries worldwide

Jungsik Noh*, Gaudenz Danuser

Supplementary methods

1. Initialization of actual time courses of new cases and current infections

The proposed framework provides estimates of time courses of actual new cases and current infections based on daily reported counts of confirmed positive tests and deaths in a particular region and published estimates of key pandemic parameters such as the infection-fatality-rate (IFR) and the mean duration from infection to death and recovery. For the purpose of this study data of daily confirmed cases and deaths for countries and U.S. states were taken from the repository by the Center for Systems Science and Engineering (CSSE) at Johns Hopkins University (1), and the COVID Tracking Project (2), respectively. Upon availability of other more granular data sets, the proposed framework can also be applied to smaller populations. The data of the country population as of July 2019 was from the United Nations (3), and the population data for U.S. states as July 2019 was from the U.S. Census Bureau (4).

To begin with, the counts of daily confirmed cases and deaths were averaged in a 7-day rolling window to remove weekend effects, which tend to yield systematic drops in the Saturday and Sunday values. To obtain initial guesses on actual counts of daily new cases and recoveries, we first combined the daily death counts and the IFR estimate ($IFR_0 = 0.66\%$) that was presented by Verity et al. (5) as follows: Let $AC_t, CC_t, I_t, D_t, R_t$ denote the number of actual new cases, confirmed new cases, currently infected cases, new deaths, and new recoveries on day t, respectively. Using the mean duration estimates (5), we assumed that one new death on day t implied $1/IFR_0$ new actual cases on day $(t - d_1), d_1 = 18$, and $(\frac{1}{IFR_0} - 1)$ new recoveries on day $(t + d_2)$, $d_2 = 7$. From this follows

$$AC_{t-d_1}^{(0)} = \frac{1}{IFR_0} D_t, \qquad R_{t+d_2}^{(0)} = \left(\frac{1}{IFR_0} - 1\right) D_t,$$

$$I_t^{(0)} = \sum_{i=1}^{t} AC_i^{(0)} - \sum_{i=1}^{t} D_i - \sum_{i=1}^{t} R_i^{(0)}, \text{ for } t = 1, 2, \ldots, T,$$

where $I_t^{(0)}$ is the derived initial estimate of the currently infected cases on day t. These initial time courses led to two other initial estimates for daily ascertainment rates (A_t), and daily ratios of the confirmed new cases to currently infected cases, referred to as detected transmission rates (DT_t):

$$A_t^{(0)} = \frac{CC_t}{AC_t^{(0)}}, \qquad DT_t^{(0)} = \frac{CC_t}{I_{t-1}^{(0)}}.$$

Both ratio estimates displayed a common increasing trend in many countries, probably indicating that the under-ascertainment was gradually improving as the testing capacities were increasing. The common trend could be exploited to obtain better estimates of the daily ascertainment rates.

2. Expectation-maximization iterations to update latent time courses

An expectation-maximization (EM) algorithm was implemented to update the latent time courses involved in actual infections. To first extract the temporal trends of the estimated daily ascertainment rates and detected transmission rates, the two rate time courses were spline-smoothed. Since the noise-levels around the temporal trends were different depending on regional population sizes, infection dynamics, and test reporting schemes, we applied multiple levels of smoothness and selected the optimal level after the complete EM computation as discussed below. For smoothing, we applied the R (6) function *smooth.spline()*. Since the

smoothed estimates of the detected transmission rates and ascertainment rates ($DT_t^{(0)}(h), A_t^{(0)}(h)$, h is a smoothness parameter) possessed a common trend, the estimated ascertainment rates could be improved by augmenting the information from $DT_t^{(0)}(h)$. The following regression model was applied to find a functional relation from $DT_t^{(0)}(h)$ to $A_t^{(0)}(h)$:

$$A_t^{(0)}(h) = \beta_0 + \beta_1 DT_t^{(0)}(h) + \beta_2 \left\{DT_t^{(0)}(h)\right\}^2 + \epsilon_t, \text{ for } t = 1, 2, \ldots, T, \qquad \text{(Eq. 1)}$$

where the regression coefficients were estimated with constraints of $\beta_1, \beta_2 \geq 0$ using an R function *penalized()*. Then, the estimated coefficients ($\hat{\beta}_j^{(0)}, j = 0, 1, 2$) were used to update the initial ascertainment rates:

$$A_t^{(1)} = \hat{\beta}_0^{(0)} + \hat{\beta}_1^{(0)} DT_t^{(0)}(h) + \hat{\beta}_2^{(0)} \left\{DT_t^{(0)}(h)\right\}^2. \qquad \text{(Eq. 2)}$$

The above EM steps enabled iterative updates of the latent time courses on actual infections. For each smoothness parameter h, the following EM algorithm was applied to obtain converged estimates (S1 Fig).

Algorithm 1. EM iterations to update the latent time courses

Input: Daily counts of confirmed new cases and deaths (CC_t, D_t). Initial time courses of actual new cases, currently infected cases, new recoveries, ascertainment rates, and detected transmission rates ($AC_t^{(0)}, I_t^{(0)}, R_t^{(0)}, A_t^{(0)}, DT_t^{(0)}$).
Output: Converged $I_t^c, AC_t^c, A_t^c, DT_t^c$.

1: **while** $I_t^{(k)}$ did not converge **do**
2: Apply spline smoothing to obtain $DT_t^{(k)}(h), A_t^{(k)}(h)$.
3: (M-step) Update the regression coefficients, $\hat{\beta}_j^{(k)}, j = 0, 1, 2$ as in (Eq. 1).
4: (E-step) Update daily ascertainment rates, $A_t^{(k+1)}$ as in (Eq. 2).
5: Update actual new case counts by $AC_t^{(k+1)} = CC_t / A_t^{(k+1)}$.
6: Update current infection counts by $I_t^{(k+1)} = \sum_{i=1}^{t} AC_i^{(k+1)} - \sum_{i=1}^{t} D_i - \sum_{i=1}^{t} R_i^{(0)}$.
7: Update detected transmission rates by $DT_t^{(k+1)} = CC_t / I_{t-1}^{(k+1)}$.
8: **end while**

3. Optimization toward the smallest variation of daily death rates

After completing EM iterations with multiple smoothness parameters (h), the converged time courses of currently infected cases were assessed by using corresponding daily rates of deaths among the infected cases:

$$DR_t(h) = \frac{D_t}{I_{t-1}^c(h)}.$$

The framework selected the smoothness parameter value ($\hat{h}$) and the final estimate of current infections ($I_t^c(\hat{h})$) that produced the smallest coefficient of variation (CV) of $DR_t(h)$ over time. Underlying this choice is the assumption that increasing variation in daily death rates would be less plausible.

To obtain 95%-confidence intervals (CIs) of the current infection estimates, the same initialization and EM iterations were applied using the lower/upper limits of the IFR estimate and

the selected smoothness ($\hat{h}$). The workflow of the initialization, EM iterations, and calculating CIs are illustrated with the case of the U.S. time courses (S1 Fig).

4. Data and code availability

The estimates of actual new infections and currently infected cases have been updated daily for 50 countries with the most confirmed cases and 50 U.S. states since August 12, 2020, in the GitHub repository (https://github.com/JungsikNoh/COVID19_Estimated-Size-of-Infectious-Population). All code, daily updated estimates, and visualizations are publicly available in this online repository.

References

1. Dong E, Du H, Gardner L. An interactive web-based dashboard to track COVID-19 in real time. Lancet Infect Dis. 2020;20(5):533-4. Epub 2020/02/23. doi: 10.1016/S1473-3099(20)30120-1. PubMed PMID: 32087114; PMCID: PMC7159018.
2. The COVID Tracking Project. https://covidtracking.com/ [Accessed September 3, 2020].
3. World Population Prospects 2019, Online Edition. Rev. 1. [Internet]. United Nations, Department of Economic and Social Affairs, Population Division. [cited April 12, 2020]. Available from: https://population.un.org/wpp/Download/Standard/CSV/.
4. Annual Estimates of the Resident Population for the United States, Regions, States, and Puerto Rico: April 1, 2010 to July 1, 2019. 2010-2019 Population Estimates. [Internet]. United States Census Bureau, Population Division. [cited April 12, 2020]. Available from: https://www.census.gov/newsroom/press-kits/2019/national-state-estimates.html.
5. Verity R, Okell LC, Dorigatti I, Winskill P, Whittaker C, Imai N, Cuomo-Dannenburg G, Thompson H, Walker PGT, Fu H, Dighe A, Griffin JT, Baguelin M, Bhatia S, Boonyasiri A, Cori A, Cucunuba Z, FitzJohn R, Gaythorpe K, Green W, Hamlet A, Hinsley W, Laydon D, Nedjati-Gilani G, Riley S, van Elsland S, Volz E, Wang H, Wang Y, Xi X, Donnelly CA, Ghani AC, Ferguson NM. Estimates of the severity of coronavirus disease 2019: a model-based analysis. Lancet Infect Dis. 2020;20(6):669-77. Epub 2020/04/03. doi: 10.1016/S1473-3099(20)30243-7. PubMed PMID: 32240634; PMCID: PMC7158570.
6. R Core Team (2019). R: A language and environment for statistical computing: R Foundation for Statistical Computing, Vienna, Austria. Available from: https://www.R-project.org/.
