# Supplementary material for: Estimation of the fraction of COVID-19 infected people in U.S. states and countries worldwide
Source: PLoS One. 2021 Feb 8;16(2):e0246772. doi: 10.1371/journal.pone.0246772 (PMC7869996; doi:10.1371/journal.pone.0246772)
Supplement: Attachment — Submitted filename: ResponseToReviewers.docx [file pone.0246772.s004.docx]

**Response to reviewers on the manuscript, “Estimation of the fraction of COVID-19 infected people in U.S. states and countries worldwide”**

We would like to thank the editor and reviewers very much for their helpful comments and suggestions. We tried to incorporate all the suggestions and have substantially revised the previous manuscript. We think that the paper is improved as a result. In the following, please find point-to-point responses to the reviewers’ suggestions.

**Reviewer #1**

C1) Abstract: New York is 12% or 0.12%?

R1) It is 0.12% as in the first manuscript.

C2) Method: line 83: Infection-Fatality-Ratio is a better as this is not rate.

R1) Since many references have been using both of the terms, ‘Infection-Fatality-Rate’ and ‘Infection-Fatality-Ratio’, the rate should be accurate enough.

C3) For referencing some indicators like the time between onset of clinical sign to death or recovery and other indicators, I recommend to use systematic review and meta-analysis.

R1) We also hope to do so. Unfortunately, we could not find any review or meta-analysis regarding the duration from the symptom onset to death or recovery, even until now (January 2021) as far as we know. We think that such estimates require individual tracking records, which have been quite challenging to acquire due to the massive infection cases. The unavailability of such datasets or meta-analysis also indicates how much we still do not know about the actual number of infections are occurring everyday worldwide.

C4) The first paragraph of the results is better to move to the methods.

R4) Revised as suggested.

C5) The quality of the pictures and photos are very low and it is not clear the name of the countries. Please replace the photos with a photo with higher resolution.

R5) The figure files were generated at the highest resolution allowed by PLOS One. The low resolution seems to be a result of automatically combining the submitted files. In the uploaded figure files themselves, the names of the regions are clearly visible. Anyone may also visit the online repository for clear figures (<https://github.com/JungsikNoh/COVID19_Estimated-Size-of-Infetious-Population>).

C6) Please check all the text, you used the full format and abbreviation of some words in different parts of the text simultaneously. For example, you used Infection-Fatality-Rate (IFR).

R6) The non-abbreviation (Infection-Fatality-Rate) was only introduced again in the supplementary and in a supplementary figure, because they are independent documents.

**Reviewer #2**

C1) The author did not write the paper systematically, such as paragraph from method is included in the introduction, and vice versa.

R1) Revised as suggested.

C2) We suggest the author to put sentences on page 4 line 67-68 into introduction section to emphasize the novelty of the study. The current issue and condition as the background of the study (including line 85-95 on page 5) should be included in introduction section, not in method section.

R2) Revised as suggested.

C3) On Page 3, line 49-51, the author stated “more importantly, we still do not know how many individuals are currently infected in many countries and regions”. Please add more references to strengthen the sentence.

R3) As far as we know, there is no published estimate of how many individuals are currently infected. Therefore, we could not add references specifically about the currently infected cases.
C4) The Method section should be described in enough detail, so that someone else could follow the steps and replicate them if they wanted to do the same study.

R4) As addressed, details of the method were stated in the supplementary appendix. Furthermore, to replicate them, anyone can download the R codes from the addressed online repository (<https://github.com/JungsikNoh/COVID19_Estimated-Size-of-Infetious-Population>).

C5) It would be better if the author put sentences on Page 3 line 54-65 in the method section regarding the source of data.

R5) Revised as suggested.

C6) The author should put the type or design or the study clearly in method section, can be written in the beginning of the first paragraph in the method section. It may also be briefly mentioned in the introduction section.

R6) Revised as suggested.

C7) It would be better if you mention and explain about spearman rank analysis, since variables were analyzed with spearman rank.

R7) Since the spearman rank correlation is a common analysis and is simply a correlation between ranks, we kept the current sentence.

C8) The author mentioned 50 countries included in the development of the computation approach, however in the result and discussion section, the author talked mainly in the USA. Please comment further on other countries, too.

R8) Though we emphasized the case of the USA, we still presented enough data for the countries. Especially the main output in Figure 4 presents the equal amount of data for the US states and countries.

C9) Please add the reference for the sentence on page 8 line 177-179. The word “allegedly” may suggest assumption, not based on valid statistical data.

R9) “Allegedly” was a typo and was removed. That sentence is supported by the Fig 3C.

C10) The applicability of the study result needs to be stated in discussion section.

R10) Revised as suggested.

C11) In the discussion section, the author did not clearly state the strength and limitation of the study. Please describe efforts to overcome the limitation as well.

R11) We mentioned the strength and limitation of the study in the 2^nd^, 3^rd^, and 4^th^ paragraphs of the discussion.

C12) The references were not based on Vancouver style, especially no 1 and 6. Please revise in regards to citation.

R12) Revised as suggested.
